# Supplementary material for: Podocalyxin and ciliary neurotrophic factor receptor are novel components of the surfaceome of chondrogenic cells
Source: Cell Commun Signal. 2025 Dec 2;24:8. doi: 10.1186/s12964-025-02552-x (PMC12777226; doi:10.1186/s12964-025-02552-x)
Supplement: Supplementary file 1 — Additional file 1 contains Supplementary Figures S1–S8 and Supplementary Tables S1–S3, providing additional data supporting the main findings. Additional files 2–5 include: differential expression analysis and clustering results for total lysate proteomes (files 2–3) and surfaceome proteomes (files 4–5). Collectively, these materials offer expanded experimental details, data visualizations, and full quantitative proteomic datasets underlying the study. [file 12964_2025_2552_MOESM1_ESM.zip › Additional-file-1.pdf]

Supporting Information

Podocalyxin and ciliary neurotrophic factor receptor are novel components of the surfaceome of chondrogenic cells

Patrik Kovács, Peter Brazda, Tibor Hajdú, Boglárka Harsányi, Krisztián Juhász, Roland Takács, Judit Vágó, Clare Coveney, David J. Boocock, Csaba Matta

Contents

Figure S1.	Volcano plots showing differentially expressed proteins (DEPs) between consecutive time points, and between undifferentiated (day 1) and mature/hypertrophic (day 15) chondrogenic micromass cultures (total lysates).
Figure S2.	Protein-protein interaction (PPI) analysis of the 6 clusters in total cell lysates using STRING based on the top 20 (hub) proteins. In each network, the size of the circles represented the degree of value: larger circles represent proteins with more connections (higher degree), meaning they interact with more other proteins in the network.
Figure S3.	Volcano plot highlighting differentially expressed proteins between consecutive time points, and between Day 1 (undifferentiated) and Day 15 (mature/hypertrophic) chondrogenic micromass cultures after AOB-enrichment.
Figure S4.	Protein-protein interaction (PPI) analysis of the 4 clusters in surfaceome samples using STRING. In each network, the size of the circles represented the degree of value: larger circles represent proteins with more connections (higher degree), meaning they interact with more other proteins in the network.
Figure S5.	Full-length uncropped blots for the western blot images presented in Figure 7.
Figure S6.	Weighted gene co-expression network analysis (WGCNA) results performed on the NGS dataset to identify clusters of highly correlated genes to CNTFR or PODXL, using culture age (time) as a trait.
Figure S7.	Transcript and protein expression patterns for the closest 25 interactants, as well as WGCNA co-expression networks, and enriched gene ontology (GO) terms in the MEs for CNTFR (A) and PODXL (B).
Figure S8.	In a single-cell atlas of healthy cartilage tissue, we identified eight chondrocyte clusters and systematically annotated their cellular identities (upper panel). Subsequent analysis revealed the expression patterns of CNTFR and PODXL across these chondrocyte subpopulations (lower panel).
Table S1.	Forward and reverse primer sequences, gene accession numbers and amplicon length for each primer pair employed in this study.
Table S2.	Comparative analysis of all total lysate and surfaceome-enriched samples, which revealed limited overlap, with only approx. 30% of proteins identified in both fractions.
Table S3.	Relative distribution of GO functional classification of the identified surface proteins during chondrogenic differentiation in micromass cultures.

Figure S1. Volcano plots showing differentially expressed proteins (DEPs) between consecutive time points, and between undifferentiated (day 1) and mature/hypertrophic (day 15) chondrogenic micromass cultures (total lysates). Cutoff values: $\log_2$ fold change > 1 (*x*-axis) and adjusted *p*-value < 0.05 (*y*-axis). Red dots signify proteins that were upregulated in later time points, whereas blue signifies proteins downregulated in later time points.

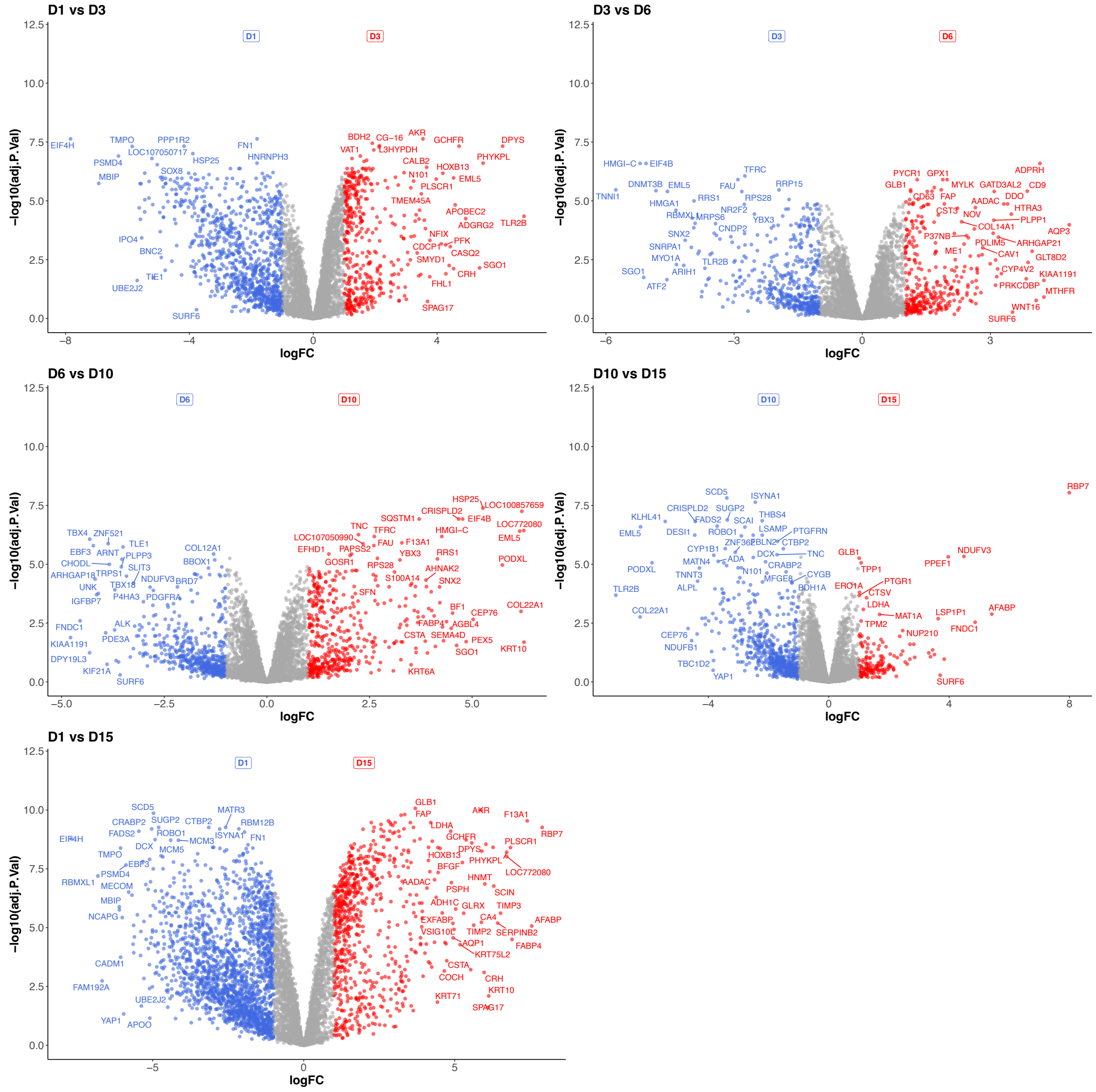

Figure S2. Protein-protein interaction (PPI) analysis of the 6 clusters in total cell lysates using STRING based on the top 20 (hub) proteins. In each subnetwork, the size of the circles represented the degree of value: larger circles represent proteins with more connections (higher degree), meaning they interact with more other proteins in the network.

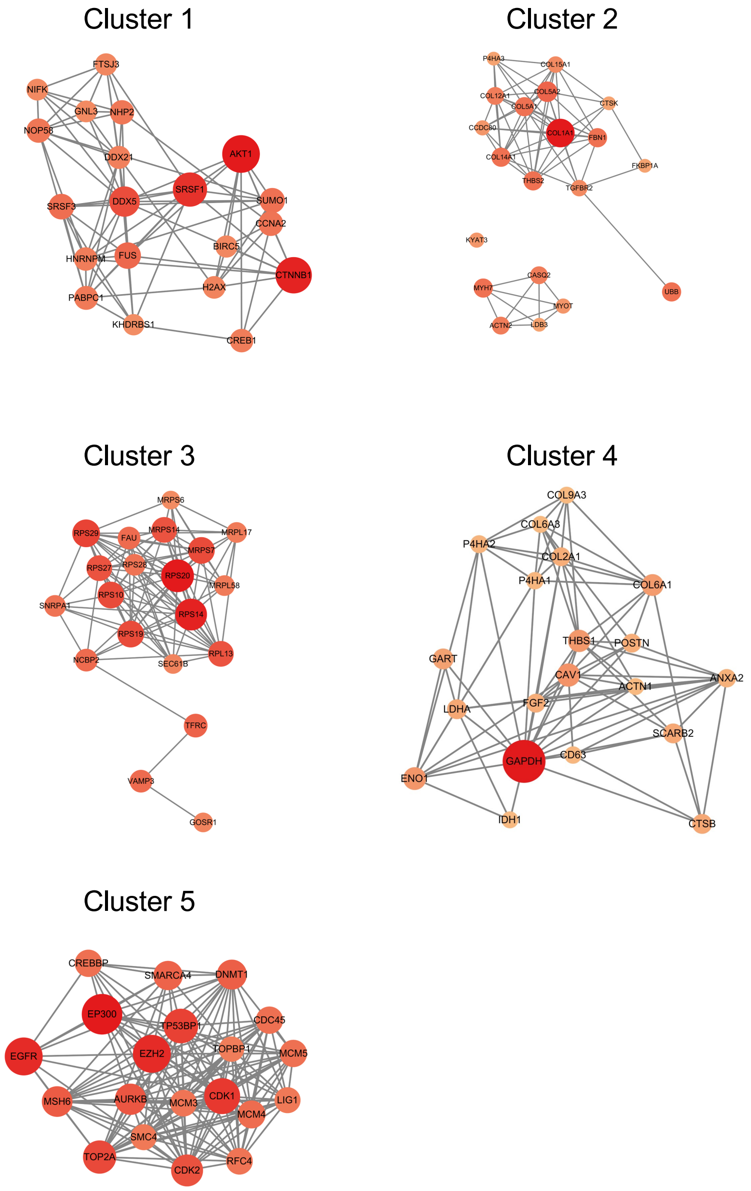

*Figure S3.*Volcano plot highlighting differentially expressed proteins between consecutive time points, and between Day 1 (undifferentiated) and Day 15 (mature/hypertrophic) chondrogenic micromass cultures after AOB-enrichment. Cutoff values: $\log_2$ fold change > 1 (horizontal axis) and adjusted *p*-value < 0.05 (vertical axis). Red dots signify proteins that were upregulated in later time points, whereas blue signifies proteins downregulated in later time points.

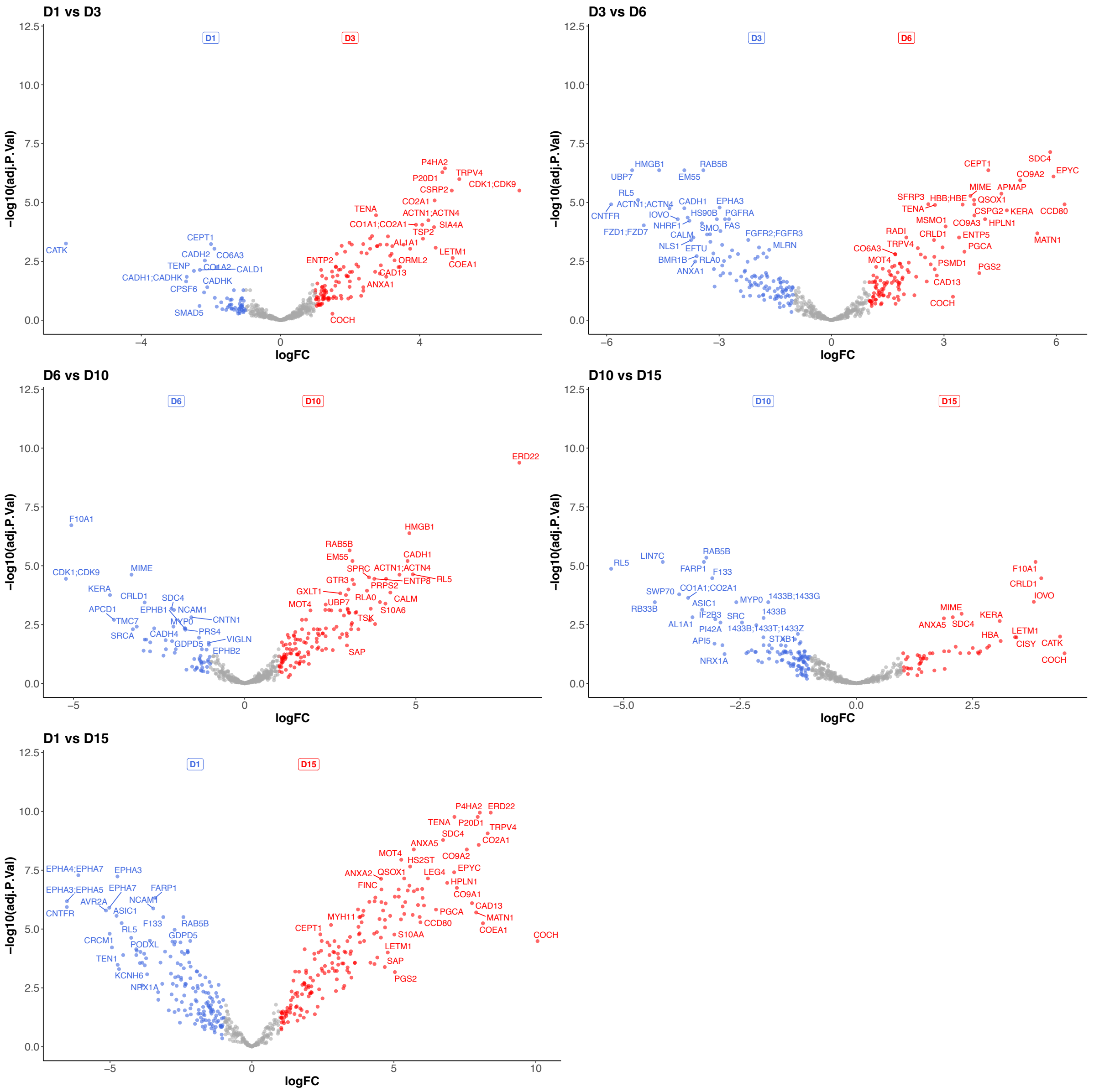

Figure S4. Protein-protein interaction (PPI) analysis of the 4 clusters in surfaceome samples using STRING. In each network/subnetwork, the size of the circles represented the degree of value: larger circles represent proteins with more connections (higher degree), meaning they interact with more other proteins in the network.

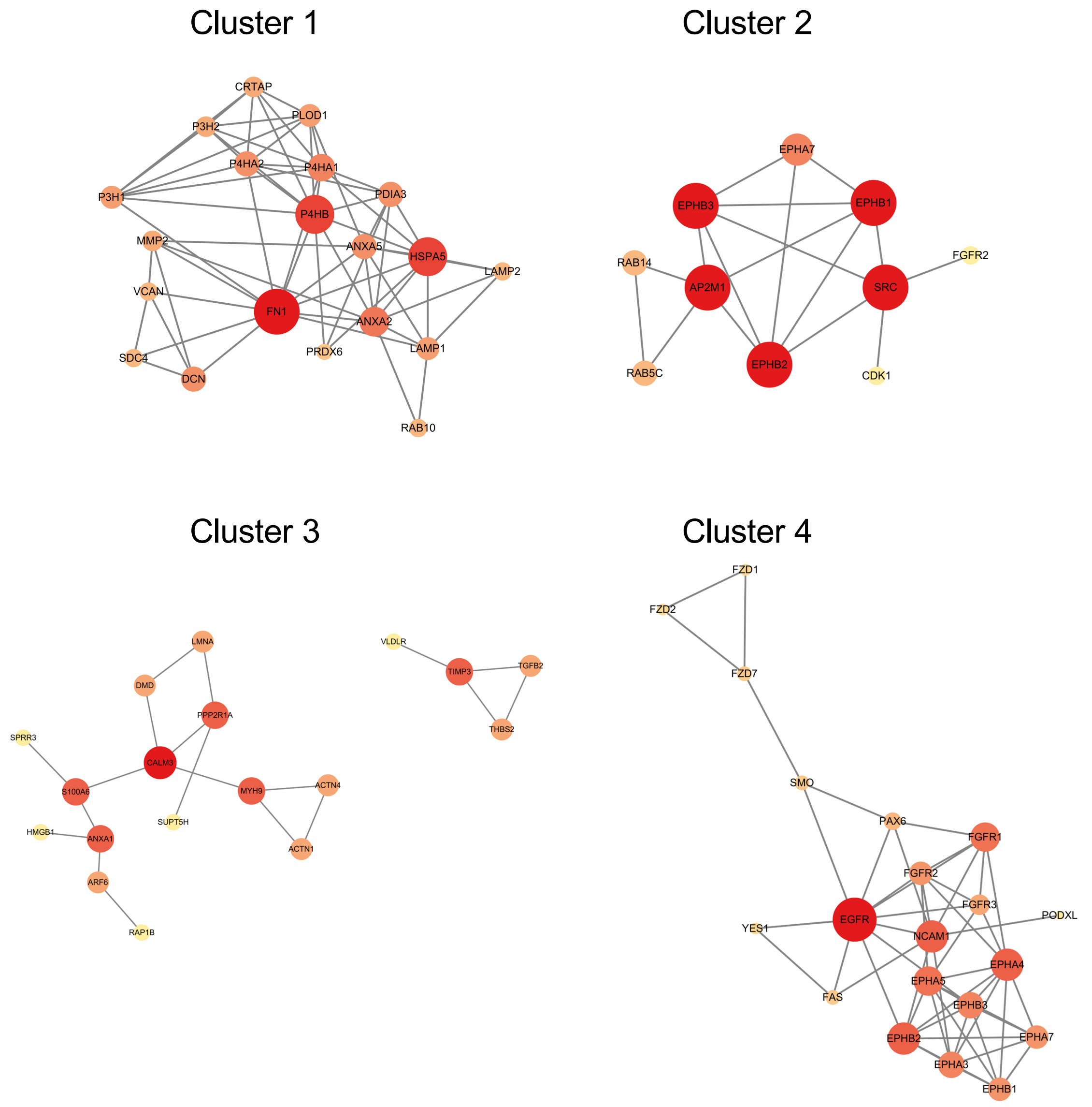

Figure S5. Full-length uncropped blots for the western blot images presented in Figure 7.

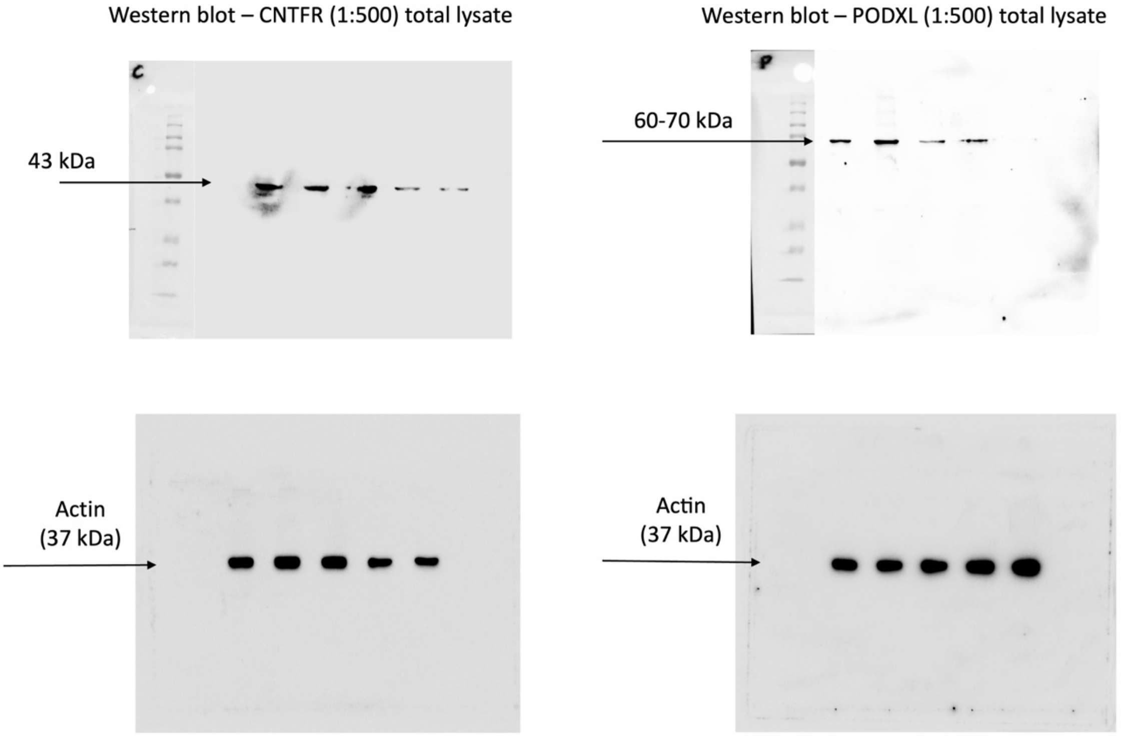

Figure S6. Weighted gene co-expression network analysis (WGCNA) results performed on the NGS dataset to identify clusters of highly correlated genes to CNTFR or PODXL, using culture age (time) as a trait. For CNTFR, the turquoise module eigengene (ME) was showing the highest degree of correlation; for PODXL, the black ME showed the highest correlation to the trait.

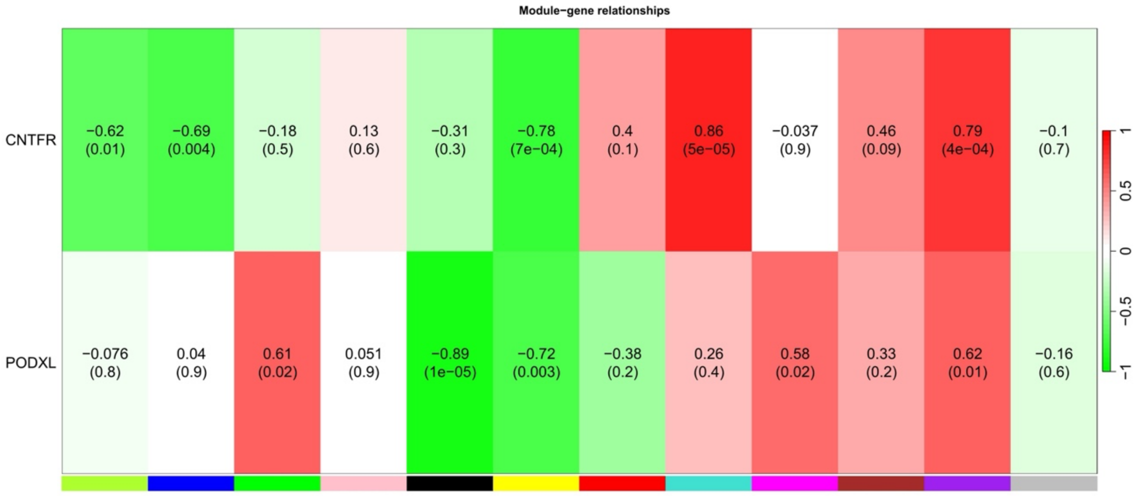

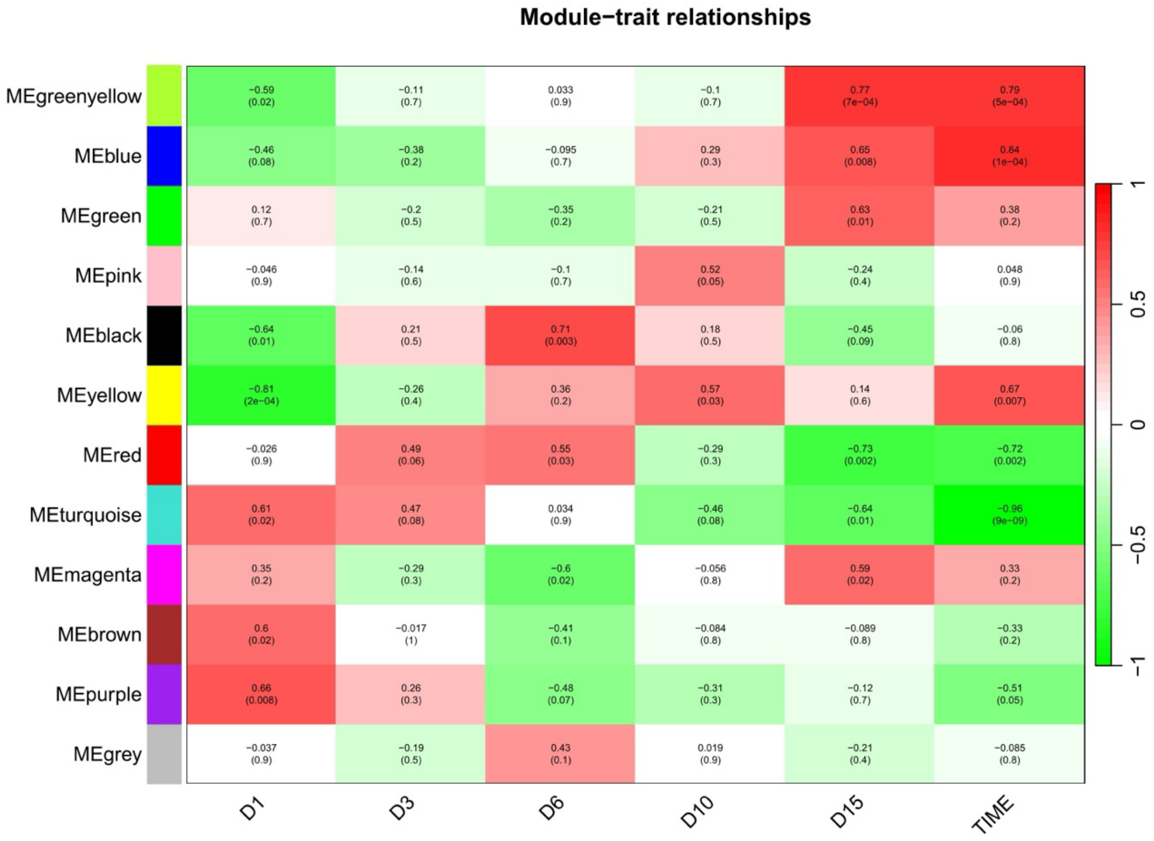

Figure S7. Transcript and protein expression patterns for the closest 25 interactants, as well as WGCNA co-expression networks, and enriched gene ontology (GO) terms in the MEs for CNTFR (A) and PODXL (B).

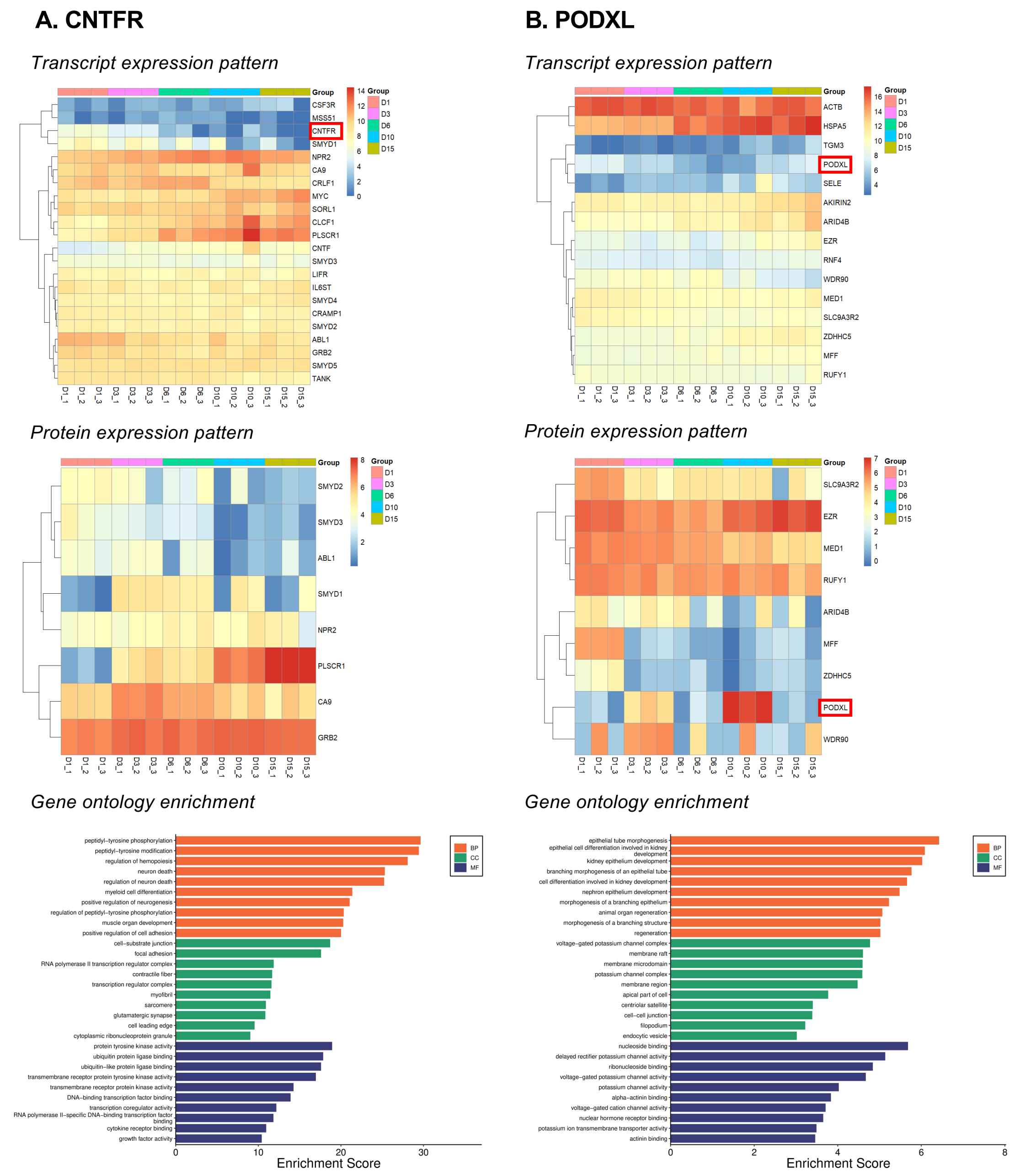

Figure S8. The single-cell atlas of healthy cartilage tissue integrates data from two public datasets: cartilage samples derived from the ankle joints of five healthy individuals (GSE216578), and healthy knee joint cartilage specimens obtained from three individuals in dataset GSE255460. Data integration and analytical processing were performed using the Seurat R package. In the single-cell atlas of healthy cartilage tissue, we identified eight chondrocyte clusters and systematically annotated their cellular identities (upper panel). Subsequent analysis revealed the expression patterns of CNTFR and PODXL across these chondrocyte subpopulations (lower panel). Notably, CNTFR demonstrated predominant expression enrichment in both effector chondrocytes (EC) and homeostatic chondrocytes (HomC). This expression signature suggests potential functional regulation of CNTFR during chondrocyte differentiation stages. PODXL demonstrated much weaker expression profile throughout chondrocyte populations, perhaps with somewhat higher transcript levels in pre-hypertrophic chondrocyte (Pre-HTC) subpopulations.

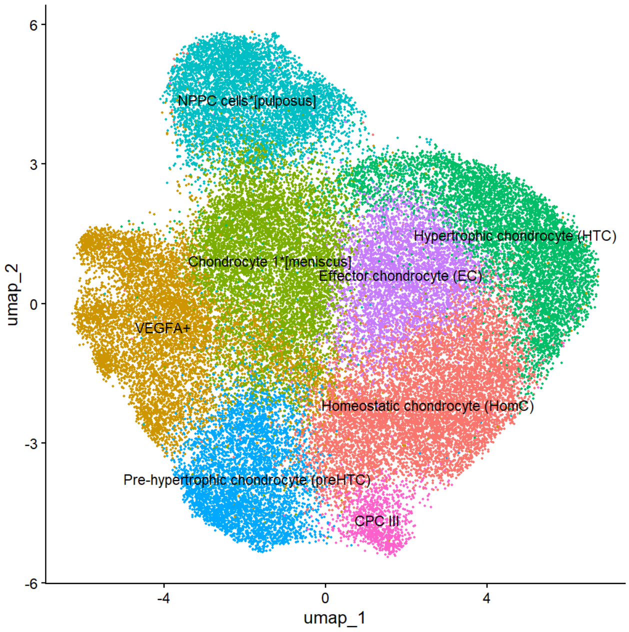

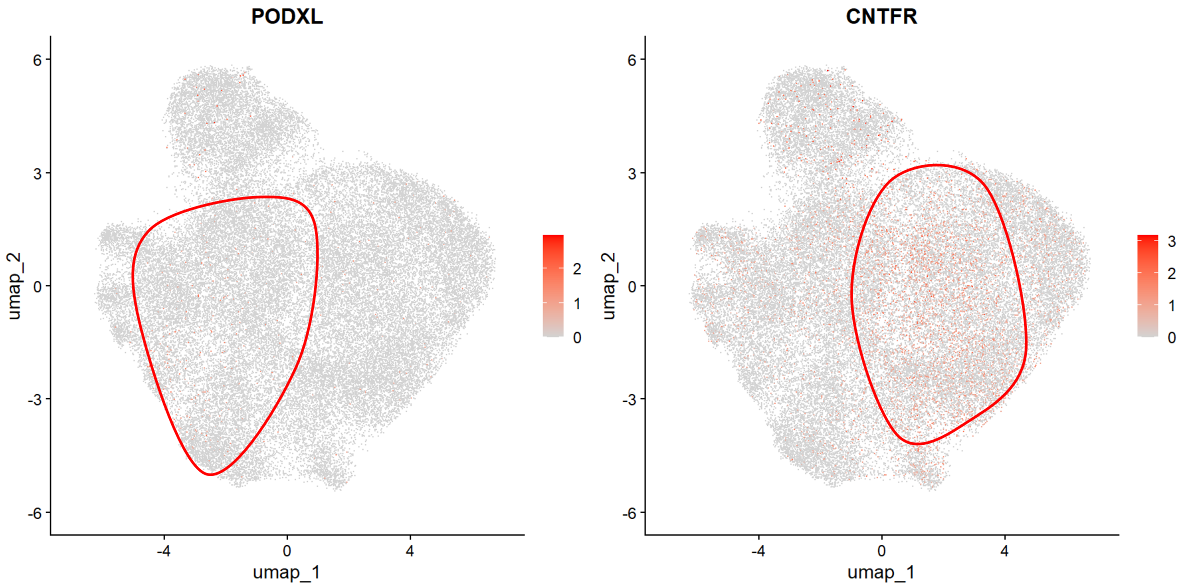

The dot plot below shows that CNTFR is highly expressed mainly in Homeostatic chondrocytes (HomC) and Effector chondrocytes (EC), while PODXL is predominantly expressed in Regulatory chondrocytes (RegC) and Pre-hypertrophic chondrocytes (preHTC).

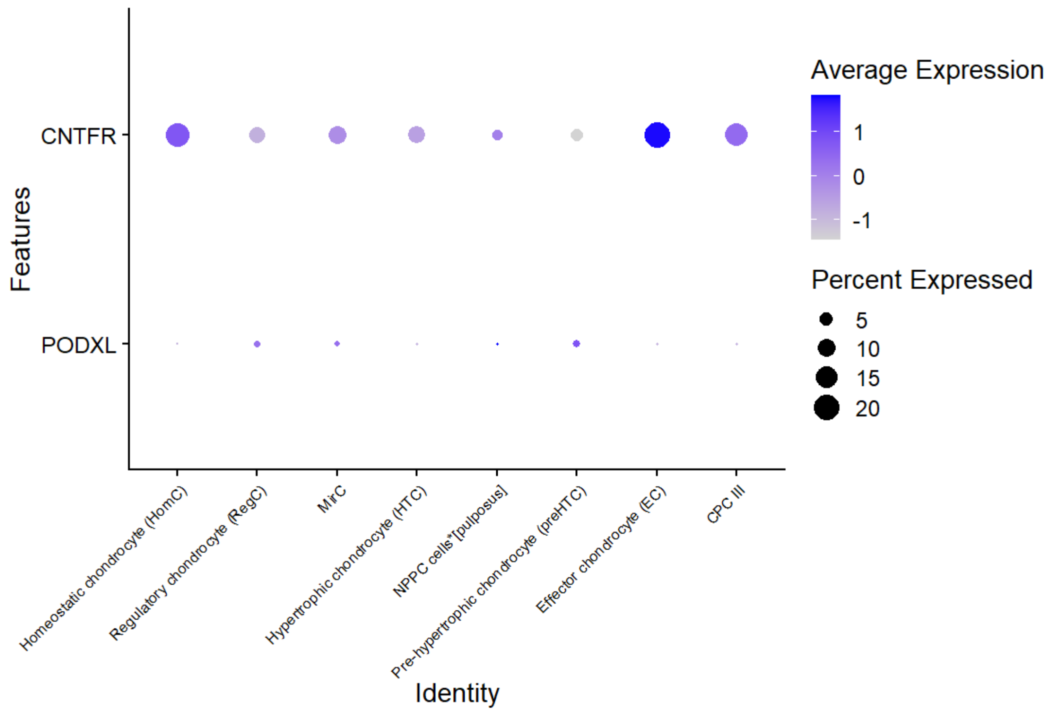
Average Expression
1
0
-1
Percent Expressed
5
10
15
20
CNTFR
PODXL
Features
Homeostatic chondrocyte (HomC)
Regulatory chondrocyte (RegC)
MirC
Hypertrophic chondrocyte (HTC)
NPPC cells*[pulposus]
Pre-hypertrophic chondrocyte (preHTC)
Effector chondrocyte (EC)
CPC III
Identity

Table S1. Forward and reverse primer sequences, gene accession numbers and amplicon length for each primer pair employed in this study.

Gene symbol, species	Accession number	Primer sequence	Product length (bp)
RPS7 *(Gallus gallus)*	XM_004940515.4	FW: CGGAGTGCCGCGAAAGG<br>REV: TGTGATGTTCAACTCCCGCA	157
YWHAZ *(Gallus gallus)*	NM_001031343	FW: GTTCCCTTGCAAAAACGGCT<br>REV: GAGGCAGACGGAAGTTGGAA	199
PPIA *(Gallus gallus)*	NM_001166326.1	FW: GAGCTCTTCGCTGACAAGGT<br>REV: GCGTAAAGTCACCACCCTGA	139
HPRT1 *(Gallus gallus)*	NM_204848.1	FW: TGGTGGGGATGACCTCTCAA<br>REV: TCCAACAAAGTCTGGCCGAT	190
RPL4 *(Gallus gallus)*	NM_001007479	FW: TGTTTGCCCCAACCAAGACT<br>REV: CTCCTCAATGCGGTGACCTT	137
RPL13 *(Gallus gallus)*	NM_204999.1	FW: GGCCCGTGTTATCTCAGAGG<br>REV: CCGCTTCTTTGGCACGTTTT	113
SOX9 *(Gallus gallus)*	NM_204281.1	FW: TTTCCGAGACGTGGACATCG<br>REV: GTACCGCTGTAGGTGGTGAC	150
ACAN *(Gallus gallus)*	NM_204955	FW: AGCAGTAGATGCACTGGGAC<br>REV: GCCAGGTCGATCTCACACAG	153
COL2A1 *(Gallus gallus)*	NM_204426.1	FW: GGGACCTCAAGGCAAAGTCG<br>REV: TTCCAGGCTCACCATTAGCG	140
COL1A1 *(Gallus gallus)*	NM_001396622.1	FW: TACTGCAACATGGAGACGGG<br>REV: CCGCCGTACTCAAACTGGAA	152
RUNX2 *(Gallus gallus)*	NM_204128	FW: GAGTCAGATTACAGACCCCAGG<br>REV: AAATGGGCCCAGCTCGGAA	199
CNTFR *(Gallus gallus)*	XM_046934809.1	FW: ATCACGGATGCCTATGCTGG<br>REV: GTGCTCGTCGTCTCTGTGAT	164
PODXL *(Gallus gallus)*	XM_046906716.1	FW: CATGTCATTGTGCATCGCGT<br>REV: TAGGTGACGTTGGTGATGCC	101

Table S2. Comparative analysis of all total lysate and surfaceome-enriched samples, which revealed limited overlap, with only approx. 30% of proteins identified in both fractions.

Sample ID	Surfaceome only	Total lysate only	Overlap	Overlap %
D1_1	289	5016	84	22.52%
D1_2	305	5010	87	22.19%
D1_3	312	5013	86	21.61%
D3_1	317	4944	93	22.68%
D3_2	335	4995	100	22.99%
D3_3	328	4998	98	23.00%
D6_1	305	5004	94	23.56%
D6_2	326	5000	100	23.47%
D6_3	303	4980	89	22.70%
D10_1	338	4900	100	22.83%
D10_2	309	4986	95	23.51%
D10_3	336	4997	101	23.11%
D15_1	326	5001	99	23.29%
D15_2	306	5008	92	23.12%
D15_3	329	4950	98	22.95%

Table S3. Relative distribution of GO functional classification of the identified surface proteins during chondrogenic differentiation in micromass cultures

	Day 1	Day 3	Day 6	Day 10	Day 15
Receptors	28%	29%	27%	28%	28%
Enzymes	33%	31%	32%	32%	31%
Transporters	21%	21%	19%	19%	20%
Adhesion proteins	24%	23%	23%	23%	23%
Miscellaneous	20%	24%	25%	25%	26%
